# Association of ovarian inflammation and hormonal dysregulation: a candidate mechanism underlying polycystic ovary syndrome

**DOI:** 10.1186/s13048-026-02218-3

**Published:** 2026-08-04

**Authors:** Shaoyu Hao, Jing Pan, Qi Zhao, Yisi A., Haijun Li

**Affiliations:** 1 College of Veterinary Medicine, Inner Mongolia Agricultural University, Hohhot, 010018 P.R. China; 2 Key Laboratory of Animal Embryo and Development Engineering of Autonomous Region Universities, Inner Mongolia Agricultural University, Hohhot, 010018 P.R. China; 3 Inner Mongolia Key Laboratory of Veterinary Fundamentals and Disease Control of Herbivorous livestock, Hohhot, 010018 P.R. China; 4 Department of Neurology, The Second Affiliated Hospital of Inner, Mongolia Medical University, Hohhot, 010030 P.R. China

**Keywords:** Polycystic ovary syndrome, Granulosa cells, RNA-seq, Inflammation, Hormonal imbalance

## Abstract

**Background:**

Polycystic ovary syndrome (PCOS) is a common endocrine disorder characterized by female infertility, menstrual irregularities, and hormonal imbalance, leading to impaired reproductive health. Hyperandrogenemia is considered a central endocrine feature and a key contributor to PCOS pathophysiology.

**Results:**

A dehydroepiandrosterone (DHEA)-induced mouse model was established to investigate transcriptomic alterations in granulosa cells (GCs) and their association with endocrine and inflammatory changes in PCOS. Model validation was performed using histological analysis (H&E staining), estrous cycle analysis, Western blotting of estrogen receptors (ERα and ERβ), and serum hormone profiling by ELISA. The PCOS model exhibited typical phenotypes, including polycystic ovarian morphology, estrous cycle arrest, and elevated testosterone (T), luteinizing hormone (LH), and LH/FSH ratios. Increased ERα expression was observed in ovarian GCs. RNA sequencing (RNA-seq) identified 161 differentially expressed genes, enriched in TNF signaling, NF-κB signaling, and steroid hormone biosynthesis pathways. In vitro, T-treated primary GCs showed increased ERα/ERβ ratio and enhanced pro-inflammatory signaling, with suppression of anti-inflammatory markers, consistent with in vivo findings. Overall, these findings support an association between endocrine alterations and inflammatory responses in ovarian GCs during PCOS.

**Conclusions:**

In conclusion, inflammatory factor expression in GCs was significantly associated with reproductive hormone dysregulation in PCOS, suggesting a potential hormone–inflammation interaction axis in the pathophysiology of PCOS.

**Graphical abstract:**

**Figure:**
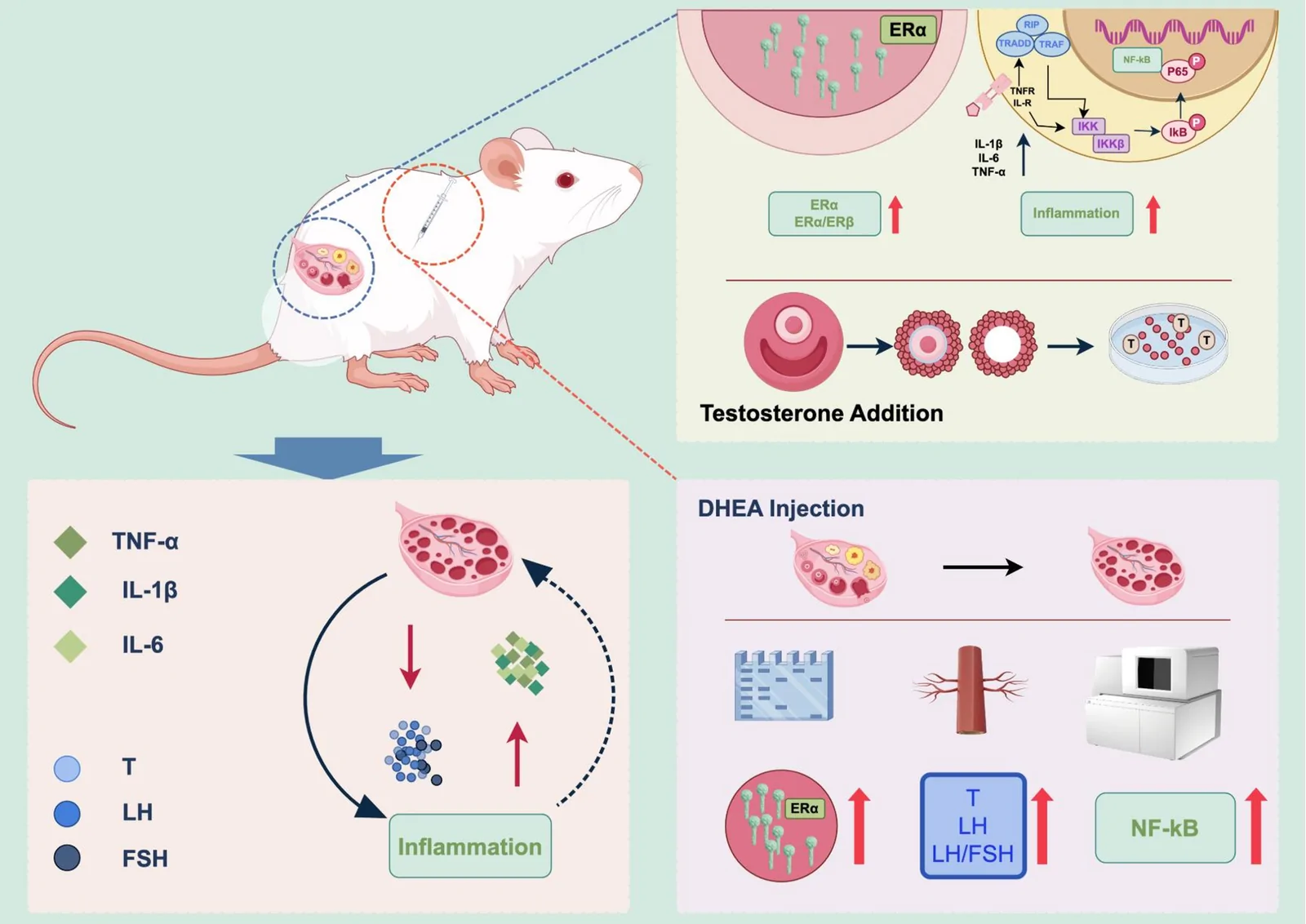

**Supplementary Information:**

The online version contains supplementary material available at https://doi.org/10.1186/s13048-026-02218-3.

## Background

Polycystic ovary syndrome (PCOS) is one of the most common endocrine disorders affecting women of reproductive age and is characterized by hyperandrogenism, ovulatory dysfunction, and polycystic ovarian morphology [1]. Unraveling the intricate pathophysiological mechanisms underlying PCOS is crucial to identifying more efficacious therapeutic approaches [2]. Hyperandrogenism is widely recognized as a hallmark endocrine abnormality of PCOS and is closely associated with impaired follicular development, endocrine imbalance, and reduced fertility [3]. Among the available experimental models, the dehydroepiandrosterone (DHEA)-induced mouse model reproduces many reproductive and endocrine characteristics of human PCOS and has therefore been extensively used to investigate its underlying molecular mechanisms [4].

Granulosa cells (GCs) are essential for follicular growth, steroid hormone synthesis, and oocyte maturation, and their dysfunction is considered a key event in the pathogenesis of PCOS [5–8]. In addition to androgen excess, increasing evidence indicates that estrogen signaling is altered in PCOS. In addition, ERα expression was significantly higher in ovarian GCs of PCOS rats and sheep than in normal controls, while ERβ expression showed the opposite trend [9, 10]. ERβ is the predominant estrogen receptor in normal GCs, whereas Studies have shown that ERα expression is also abnormally increased in the GCs of PCOS patients, and the ERα/ERβ ratio is positively correlated with AMH content [11]. These findings suggest that the ERα/ERβ ratio reflects altered ovarian endocrine signaling and may serve as an additional indicator for evaluating whether the DHEA-induced mouse model recapitulates endocrine characteristics observed in human PCOS.

RNA sequencing (RNA-seq) utilizes its high-throughput transcriptome profiling capabilities to identify key pathogenic genes, analyze core signaling pathways, and investigate epigenetic abnormalities in the pathological research of PCOS. In a study employing single-cell RNA-seq, Harris et al. [12] analyzed ovarian theca cells isolated from PCOS patients and normal individuals, revealing differences in gene expression related to cholesterol acquisition and steroidogenesis. However, relatively few studies have utilized RNA-seq to characterize mouse ovarian GCs. Moreover, although hyperandrogenism has been shown to induce endoplasmic reticulum stress, apoptosis, and functional impairment in GCs [13], the transcriptomic alterations associated with endocrine imbalance and their relationship with inflammatory responses in GCs remain poorly understood.

Despite growing evidence linking endocrine dysregulation and inflammation in PCOS, their transcriptomic association in ovarian GCs remains unclear. We hypothesized that hyperandrogenism-associated endocrine dysregulation is associated with transcriptomic remodeling and inflammatory responses in GCs. To test this hypothesis, we performed RNA-seq of GCs from a DHEA-induced PCOS mouse model and validated inflammation-related changes using a testosterone (T)-treated primary GC model.

## Methods

### Animal euthanasia

All animal procedures were carried out in strict adherence to the guidelines ratified by the Research Ethics Committee of Inner Mongolia Agricultural University, China (approval ID: NND2022002) and the AVMA Guidelines for the Euthanasia of Animals. Prior to sacrifice, female mice were anaesthetized via injection of sodium pentobarbital (50 mg/mL) at a dose of 100 mg/kg body weight. Euthanasia was then performed by cervical dislocation.

### Mouse treatment

Specific pathogen‑free (SPF) female ICR mice (21 days of age) used in this study were purchased from Si Pei Fu (Beijing) Biotechnology Co., Ltd. All animals were housed under a 12‑h light/12‑h dark cycle (lights on 6:00, lights off 18:00). Mice were randomly assigned to two groups: a normal control group and a DHEA-treated group ICR mice exhibit excellent adaptability, a sturdy physique, high reproductive capacity, rapid growth, a docile temperament, and ease of experimental handling [14–16]. A mixture of absolute ethanol 1∶sesame oil 9 (v/v) was used as the solvent for preparing the DHEA (GLPBIO, GC11070, USA) solution. DHEA group mice were given daily DHEA injections (6 mg/100 g BW, 0.1 mL), and the control group was administered an equivalent volume of absolute ethanol‑sesame oil vehicle [17]. Beginning at 22 days of age, all mice received once-daily subcutaneous injections for 21 consecutive days. Blood samples and ovarian tissues were harvested 2 days after the final injection.

### Estrous cycle determination

Vaginal smears were collected daily (*n* = 3/group) for 10 days following 21 days of DHEA treatment, and estrous cycle stages were identified microscopically after gentian violet staining according to the main cell type. Nucleated epithelial cells in proestrus, cornified squamous epithelial cells in estrus, mixed leukocytes, cornified epithelial cells and nucleated keratinocytes in metestrus, and predominantly leukocytes with plentiful mucus in diestrus [18].

### Reproductive capacity

Three females from each group with sexually mature male ICR mice at a 1:1 ratio. Litter size and the latency from pairing to the first parturition were recorded and quantified for each group.

### Enzyme-Linked Immunosorbent Assay (ELISA) for hormones

Freshly collected mouse blood was stored at 4 °C for 2 h and then centrifuged at 3000 rpm for 20 min at 4 °C, after which the supernatant was carefully collected. The serum samples were stored at − 80 °C refrigerator prior to assay. Serum concentrations of T, progesterone (P4), luteinizing hormone (LH) and follicle‑stimulating hormone (FSH) in mice were measured by ELISA (*n* = 4/group), whereas estradiol (E2) was measured in a separate cohort (*n* = 3/group) using the corresponding ELISA kits (Elabscience Biotechnology Co., Ltd.).

### Morphology and histochemistry

To assess polycystic ovarian morphology, ovaries from three mice per group (*n* = 3/group) were evaluated by hematoxylin and eosin (H&E) staining.

Ovarian tissues were harvested and fixed in 4% paraformaldehyde (Solarbio, P1110) at 4 °C for 12 h, sequentially dehydrated in 70%, 95%, and 100% ethanol at room temperature for 30 min each, cleared in xylene for 30 min, and embedded in paraffin sequentially. The paraffin-embedded blocks were sectioned and stained with H&E staining. Images were captured using a Nikon C2 confocal microscope (Nikon, Tokyo, Japan). Serial sections were examined to identify corpora lutea.

### Extraction and cultivation of primary mouse GCs

Primary GCs were isolated from 21-day-old female mice (*n* = 3 independent isolations). Mice received 5 IU pregnant mare serum gonadotropin 48 h before collection. GCs were released by follicle puncture, filtered, centrifuged, and cultured at 37 °C with 5% CO₂ [19]. After 24 h, GCs were treated with T (Sigma, St. Louis, MO, USA) at 8 ng/mL (low T) or 260 ng/mL (high T) for 24 h, based on serum T levels in the DHEA-induced PCOS mouse model. Unless otherwise indicated, all in vitro experiments were performed using *n* = 3 independent biological replicates, each derived from an independent cell isolation. In this study, PCOS-like GCs were defined as primary GCs exposed to 260 ng/mL T for 24 h.

### RNA-seq analysis

Total RNA was extracted from mouse ovarian granulosa cell samples using Trizol reagent (Thermo Fisher, cat. 15596018). RNA purity and integrity were assessed via Agilent 2100 Bioanalyzer and RNA 6000 Nano Kit (Agilent, cat. 5067 − 1511). Only RNA samples with an RNA Integrity Number (RIN) > 7.0 and qualified purity were included in subsequent library construction. The mRNA was purified from 5 µg total RNA using Dynabeads Oligo (dT) (Thermo Fisher) with two purification rounds, then fragmented at 94 °C for 5 min using the Magnesium RNA Fragmentation Module (NEB, cat. E6150). Fragmented RNA was reverse-transcribed to cDNA with SuperScript™ II Reverse Transcriptase (Invitrogen, cat. 1896649), followed by synthesis of U-labeled second-stranded DNAs using E. coli DNA polymerase I (NEB, cat. M0209), RNase H (NEB, cat. M0297), and dUTP Solution (Thermo Fisher, cat. R0133). An A-base was then added to the blunt ends of each strand, preparing them for ligation to the indexed adapters. Dual-index adapters were ligated to the cDNA fragments, and size selection was conducted using AMPure XP beads. The U-labeled second-stranded DNAs were treated with heat-labile uracil-DNA glycosylase (UDG; New England Biolabs, Ipswich, MA, USA; cat. no. M0280), followed by PCR amplification of the ligated products under the following conditions: initial denaturation at 95 °C for 3 min; 8 cycles of denaturation at 98 °C for 15 s, annealing at 60 °C for 15 s, and extension at 72 °C for 30 s; and a final extension at 72 °C for 5 min. The average insert size of the final cDNA libraries was 300 ± 50 bp. Finally, 2 × 150 bp paired-end sequencing (PE150) was performed on an Illumina NovaSeq™ 6000 platform (LC-Bio Technology Co., Ltd., Hangzhou, China) for all qualified libraries were sequenced. A standard sequencing depth of 5–6 Gb clean data per sample was guaranteed to ensure sufficient coverage for transcriptomic analysis.

Raw sequencing reads were processed using Cutadapt (v1.9) to remove adapter sequences, low-quality bases, and short reads, generating high-quality clean reads. Strict quality control criteria were applied: clean reads accounted for more than 96% of raw reads, with Q20 > 99%, Q30 > 97%, and stable GC content, indicating reliable sequencing data quality. The clean reads were aligned to the mouse reference genome using HISAT2 (v2.2.1), with an overall genome mapping rate > 93% and unique mapping rate > 88% across all samples. Batch effect assessment was performed via principal component analysis (PCA), and the results showed that intragroup samples clustered tightly with no significant batch deviation, eliminating the interference of batch effects on subsequent analysis.

Based on the genome alignment results, transcript assembly of each sample was independently performed using StringTie (v2.1.6) with default parameters. GffCompare (v0.9.8) was used to annotate, compare and integrate assembled transcripts from all samples, generate a unified global transcriptome, and classify novel transcript isoforms. Transcript and gene expression levels were quantified by StringTie, and FPKM (fragment per kilobase of transcript per million mapped reads) values were calculated for standardized expression presentation of all mRNAs. The average value of FPKM values of all gene expression levels in each sample of the same group > 12, and the standard deviation of FPKM values of all gene expression levels in each sample of the same group > 91. For intergroup differential expression analysis, DESeq2 software was adopted with the median of ratios normalization strategy to eliminate the influence of sequencing depth and gene length bias. EdgeR was used for auxiliary pairwise comparison verification to improve the stability of differential screening results.

Differentially expressed genes (DEGs) between the control group (CTRL1–3) and DHEA-induced PCOS model group (DHEA-M1–3) were identified using DESeq2 with thresholds of false discovery rate (FDR, q value) < 0.05 and absolute fold change ≥ 2(|log2FC|>=1). Enrichment analyses of GO (Gene Ontology) functions and KEGG (Kyoto Encyclopedia of Genes and Genomes) pathways were further performed for the DEGs.

### Quantitative real-time PCR analysis

Quantitative real-time PCR (qRT-PCR) was used both to validate representative DEGs identified by RNA-seq and to assess the expression of inflammation-related genes in primary GCs. The cDNA was synthesized from total RNA using the PrimeScript™ RT Reagent Kit (Takara Bio, Shiga, Japan; cat. no. RR047A). Gene-specific primers used for qRT-PCR are listed in Table 1. qRT-PCR was performed on an ABI 7500 Real‑Time PCR System (Applied Biosystems) using TB Green^®^ Premix Ex Taq™ II (Takara Bio, Shiga, Japan; cat. no. RR820A). Relative gene expression was normalized to the mouse ACTIN and calculated using the 2^−ΔΔCt method.

**Table 1:** Sequence of primers

Gene	Sequence of forward primer	Sequence of reverse primer
β-Actin	ATATCGCTGCGCTGGTCG	TTCCCACCATCACACCCTGG
Tnf	TGCCTATGTCTCAGCCTCTTC	GAGGCCATTTGGGAACTTCT
Il-6	CCAGAAACCGCTATGAAGTTCC	GTTGGGAGTGGTATCCTCTGTGA
Il-1β	GCAACTGTTCCTGAACTCAACT	ATCTTTTGGGGTCCGTCAACT
Il-4	TCTCGAATGTACCAGGAGCCA	AGGACGTTTGGCACATCCA
Il-10	GGTTGCCAAGCCTTATCGGA	CACCTTGGTCTTGGAGCTTATT
Comp	ACTGCCTGCGTTCTAGTGC	CGCCGCATTAGTCTCCTGAA
Car4	TACGTGGCCCCCTCTACTG	GCTGATTCTCCTTACAGGCTCC
Nr4a1	TTGAGTTCGGCAAGCCTACC	GTGTACCCGTCCATGAAGGTG
Irf7	GAGACTGGCTATTGGGGGAG	GACCGAAATGCTTCCAGGG
Vcam1	AGTTGGGGATTCGGTTGTTCT	CCCCTCATTCCTTACCACCC
Mxra8	GCGCCGACTTGTGGATATGTA	GCCGTCGTGGAAAGCAGAA
Icam1	GTGATGCTCAGGTATCCATCCA	CACAGTTCTCAAAGCACAGCG
Mro	GCCCGGACCTTACTGGATG	GAGCGAATCTTGGGTCTGGTT
Cmtm1	ATACGTGCTATACCATCCAGGC	TGACGTGTACTAAAGGTCGCT

### Western blot

GCs total proteins were extracted using RIPA buffer (CWBIO) supplemented, with 1% protease inhibitor. Protein concentrations were measured using a BCA kit (Thermo Fisher Scientific) and diluted to a consistent concentration. Proteins were separated by 12% SDS‑PAGE and transferred to 0.45 μm PVDF membranes (Merck Millipore). Membranes were blocked with LI‑COR blocking buffer for 1 h, then with primary antibodies, including mouse anti‑α‑tubulin (1:10,000; T5168, Sigma), mouse anti-β-actin antibody (1:10,000, A5316, Sigma, ), rabbit anti-ERβ antibody (1:1000, PA1-311, Thermo Fisher Scientific), rabbit anti-ERα antibody (1:1000, PA5-16440, Thermo Fisher Scientific, ), mouse anti-IL-1β (1:1000, HA601036, Huaan, Hangzhou, China), and mouse anti-IL-6 (1:500, EM1701-45, Huaan, Hangzhou, China). Subsequently, the membranes were incubated with secondary antibodies, including donkey antirabbit (1:10,000, 926-32213 LI-COR Biosciences, Lincoln) and donkey anti-mouse (1:10,000, 926-32212, LI-COR Biosciences, Lincoln). Protein bands were visualized and scanned using the Odyssey Infrared Imaging System (LI-COR Biosciences, Lincoln). α-Tubulin and β-Actin were used as the internal reference proteins. Original images of the Western blot analyses are provided in Supplementary File S1.

### Statistical analysis

All experiments were performed with 3 independent biological replicates, each including several technical replicates, and are presented as mean ± SD (*n* ≥ 3). Raw data were screened by normality (Shapiro-Wilk test, *P* > 0.05) and equal variance (Bartlett’s test, *P* > 0.05) to select non-parametric analyses accordingly. For comparisons between independent groups, an unpaired t-test (for two groups), a one-way ANOVA (for three or more groups) and two-way ANOVA (for two factors) was used. Non-normal data were analyzed with two-tailed Mann–Whitney U (two groups) plus Tukey’s post-hoc test. GraphPad Prism 8.0 was used for graph plotting. *P < 0.05, **P < 0.01, ***P < 0.001.

## Results

### DHEA impaired mouse ovarian morphology and the estrous cycle

**Fig. 1:**
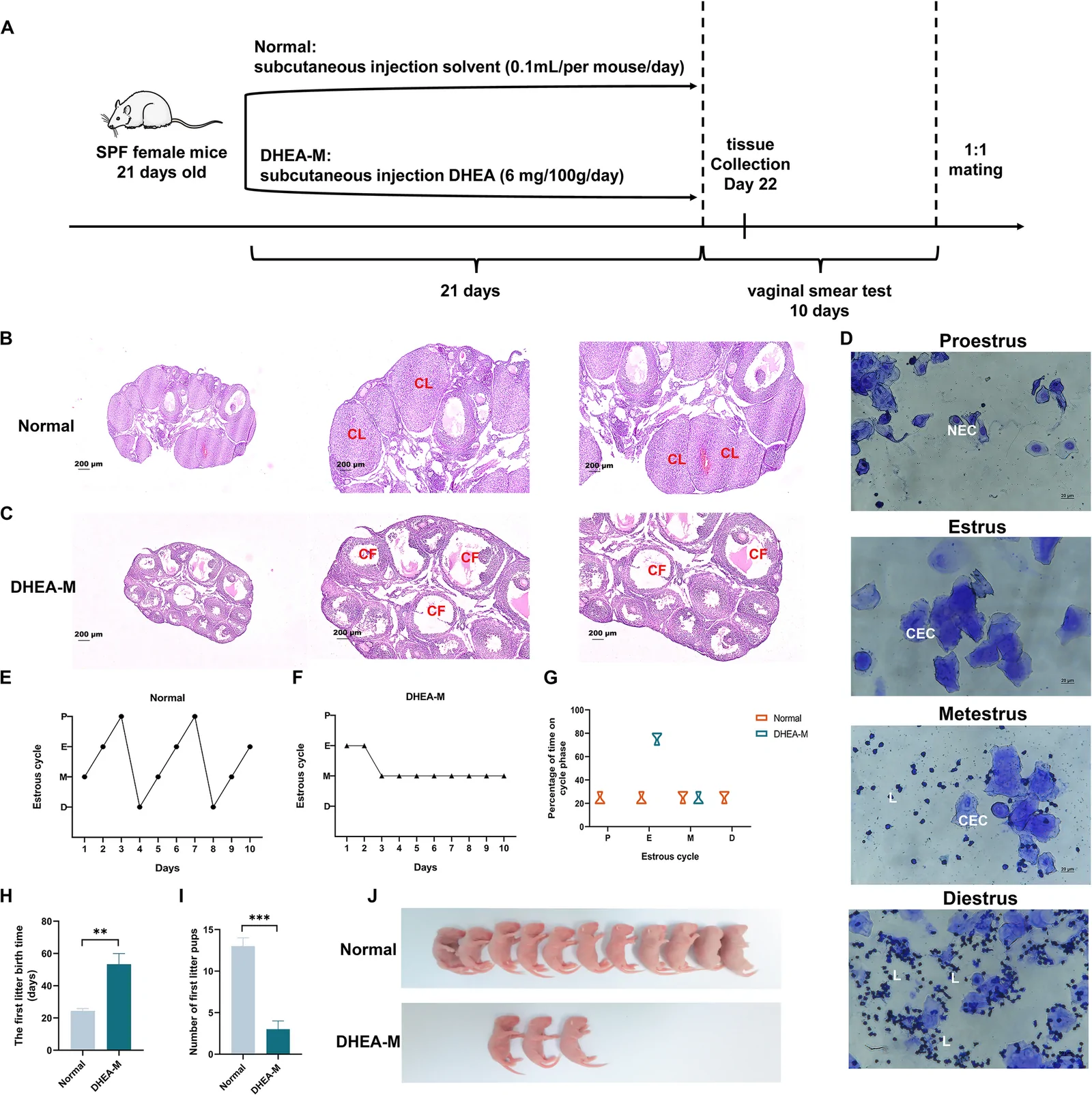
Induction and validation of the DHEA-induced PCOS mouse model. **A** The experimental procedure. Representative images of HE staining of ovaries in the normal (**B**) and DHEA-M (**C**) groups (*n* = 3). Scale bar, 200 μm. **D** Representative estrous cycles based on cytological assessment of vaginal smears (scale bar = 20 μm). Proestrus is characterized by round nucleated epithelial cells (NEC); Estrus is marked by keratinized squamous epithelial cells (CEC); Metestrus shows a mixture of CEC and leukocytes (L); Diestrus is identified by nucleated epithelial cells with a predominance of L; (**E**) 10-day estrous cycle dynamics in normal group mice (*n* = 3). (**F**) 10-day estrous cycle dynamics in DHEA-M group mice (*n* = 3). **G** Violin plot depicting 10-day estrous cycle dynamics in mice of each group. **H** The first litter birth time after the model was constructed (mean ± SD, *n* = 3, T-test). **p < 0.01. **I** Number of first litter pups (mean ± SD, *n* = 3, T-test). ***p < 0.001. **J** The morphology of mouse pups. CL: corpora lutea, CF: cystic follicle, P: Proestrus, E: Estrus, M: Metestrus, D: Diestrus

Mice were subcutaneously injected with DHEA or vehicle once daily starting from postnatal day 22, and the treatment was administered continuously for 21 consecutive days. On the day following the final injection, ovaries and serum samples were harvested, and vaginal smears were collected and examined daily for a consecutive 10-day period (Fig. 1A).

To further investigate the effects of DHEA administration on ovarian morphology in mice, histological and morphological analyses were performed on ovarian tissues, and the results showed that multiple corpora lutea in the normal group (Fig. 1B). However, antral follicles and seldom corpora lutea were observed in the PCOS group (Fig. 1C). Moreover, the GCs were arranged loosely. In summary, DHEA treatment significantly inhibits normal follicular development and promotes ovarian follicular cystogenesis.

To determine the effects of DHEA treatment on ovulation in mice, vaginal smears stained with gentian violet were employed to monitor alterations in the estrous cycle. The results demonstrated that mice in the normal group exhibited a regular estrous cycle (Fig. 1E and G), characterized by nucleated epithelial cells at proestrus, cornified squamous epithelial cells at estrus, abundant leukocytes at diestrus, and a mixture of the three cell types at metestrus. (Fig. 1D). However, mice in the PCOS group displayed a disrupted estrous cycle and remained arrested in the metestrus stage, which was dominated by a large number of leukocytes (Fig. 1F and G). After a 10-day period of sexual cycle testing, 3 female mice were randomly selected from each group and housed together with healthy adult ICR stud males at a ratio of 1:1. Compared with normal group, the interval to first-litter delivery in the PCOS group was significantly extended (Fig. 1H), and the number of pups in the first littler decreased significantly (Fig. 1I). No morphological abnormalities were observed in mouse pups (Fig. 1J). Collectively, these findings supporting the successful establishment of a PCOS mouse model.

### DHEA supplementation disrupts steroidogenesis, gonadotropin secretion and ER expression

**Fig. 2:**
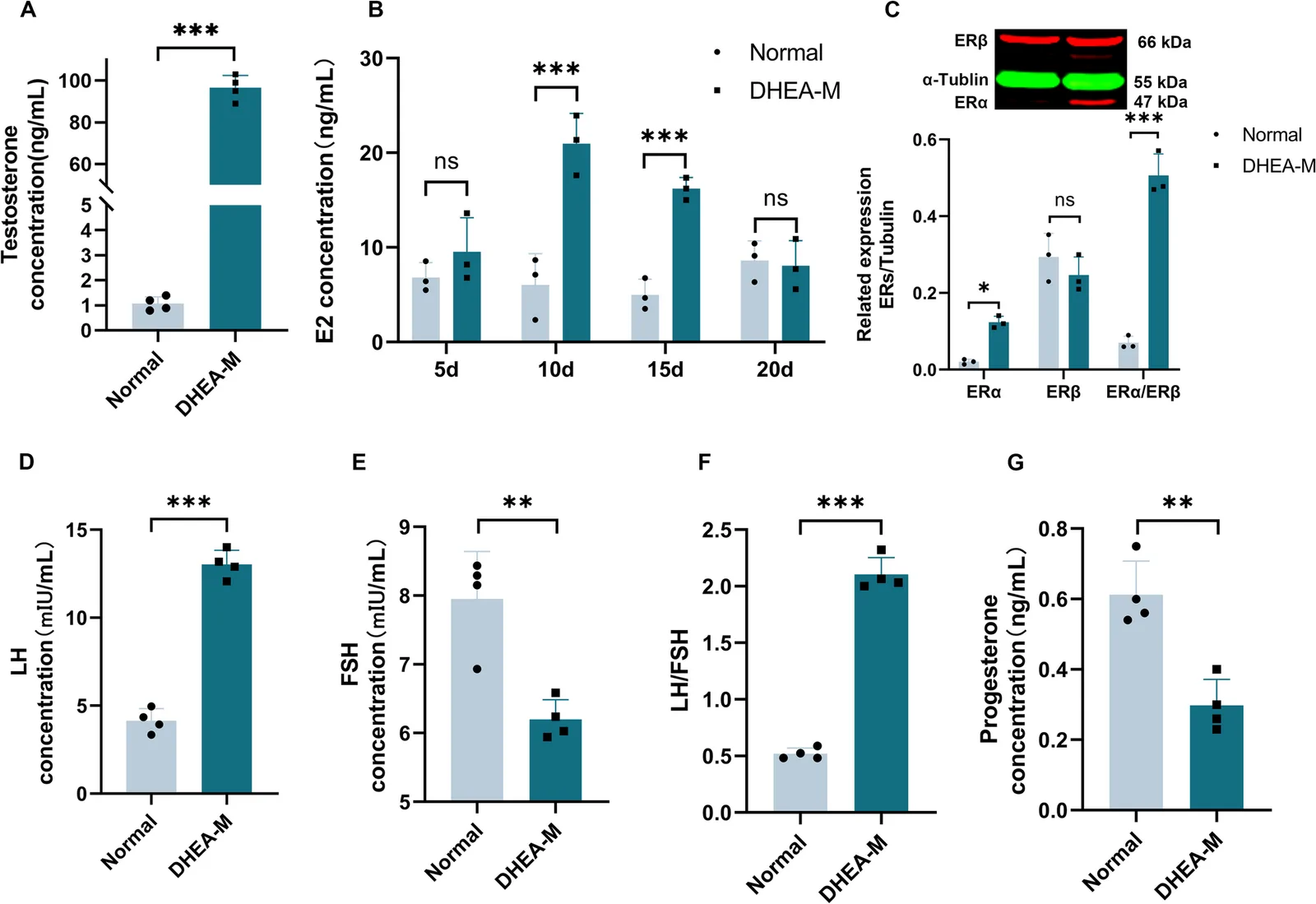
Steroidogenesis, Gonadotropins Secretion and ER Expression Changes in the PCOS model established by DHEA. **A** Changes of serum T levels in mice with PCOS induced by DHEA (mean ± SD, *n* = 4, T-test). ***p < 0.001. **B** Changes of serum E2 levels in mice with PCOS induced by DHEA (mean ± SD, *n* = 3, Two-way ANVOA). ***p < 0.001. **C** Changes of ERs protein levels in the GCs of mice with PCOS induced by DHEA (mean ± SD, *n* = 3, Two-way ANVOA). *p < 0.05, ***p < 0.001. **D** Changes of serum LH levels in mice with PCOS induced by DHEA (mean ± SD, *n* = 4, T-test). ***p < 0.001. **E** Changes of serum FSH levels in mice with PCOS induced by DHEA (mean ± SD, *n* = 4, T-test). *p < 0.05. **F** Changes of serum LH/FSH ratio in mice with PCOS induced by DHEA (mean ± SD, *n* = 4, T-test). ***p < 0.001. **G** Changes of serum progesterone levels in mice with PCOS induced by DHEA (mean ± SD, *n* = 4, T-test). **p < 0.01

To further characterize the endocrine features of the DHEA-induced PCOS model, serum reproductive hormones and ER expression in ovarian GCs were evaluated. Compared with the normal group, DHEA-treated mice exhibited significantly increased serum T and LH levels (*p* < 0.001, Fig. 2A and D), accompanied by an elevated LH/FSH ratio (*P* < 0.001; Fig. 2F). In contrast, FSH and P4 levels were significantly decreased (*p* < 0.01, Fig. 2E; *p* < 0.01, Fig. 2G), while E2 levels were significantly increased on day10 and day 15(*p* < 0.001, Fig. 2B). These endocrine changes were consistent with the hormonal characteristics of PCOS.

Western blot analysis further showed that ERα protein expression was significantly increased in ovarian GCs from DHEA-treated mice (*P* < 0.05, Fig. 2C), whereas no significant difference in ERβ protein expression was observed (*P* > 0.05, Fig. 2C). Consequently, the ERα/ERβ ratio was significantly elevated compared with the normal group (*P* < 0.001, Fig. 2C). These findings indicate that the DHEA-induced mouse model reproduced endocrine alterations and changes in estrogen receptor signaling reported in PCOS, providing a foundation for subsequent transcriptomic and inflammation-related analyses.

### Transcriptional landscapes of PCOS GCs

**Fig. 3:**
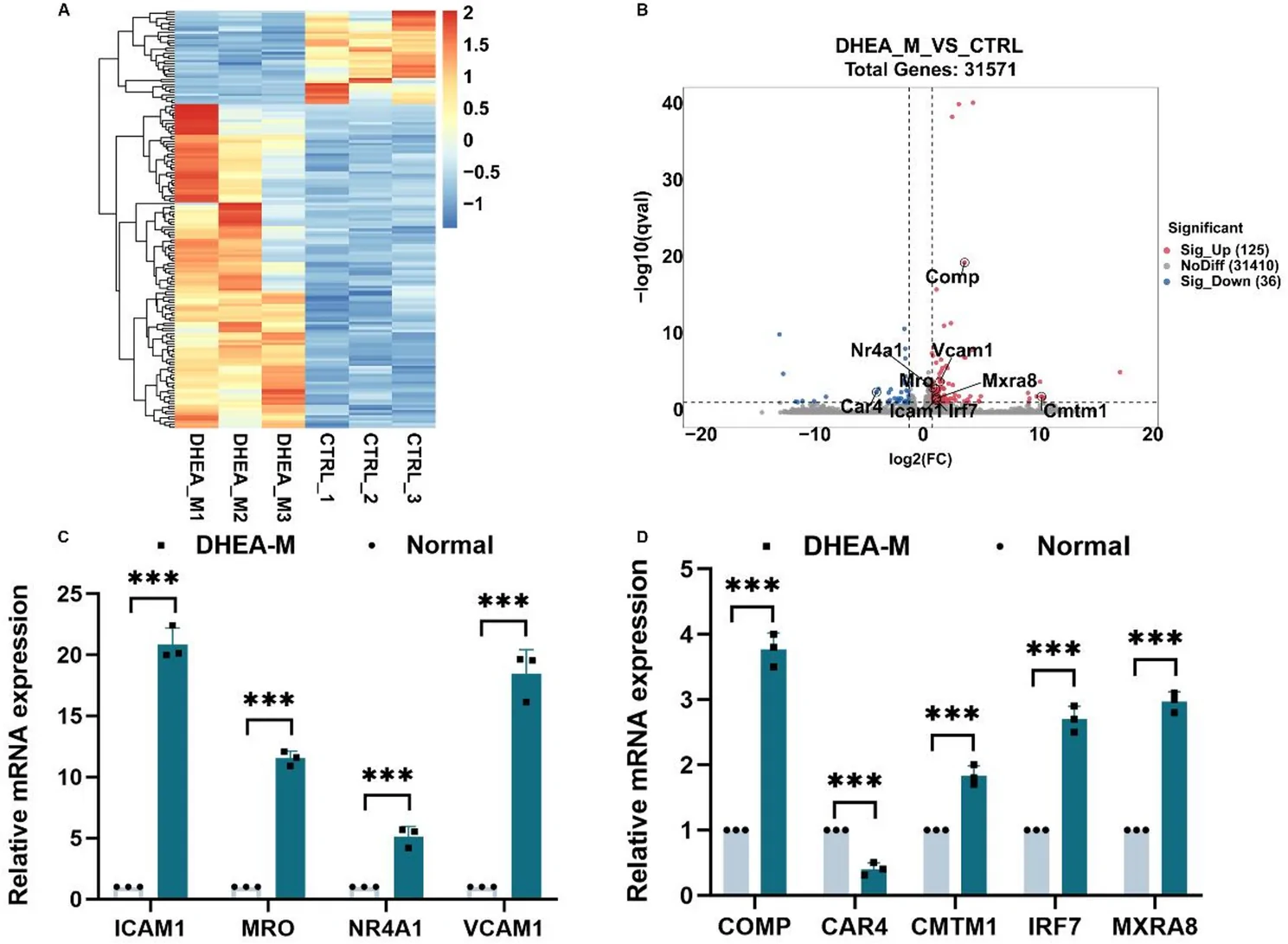
Profiling of the transcriptomic landscape in mouse PCOS GCs. **A** Heatmap of DEGs in the CTRL and DHEA-M. **B** Volcano plot showing transcriptomic landscapes in the CTRL and DHEA-M. **C**, **D** The validation of RNA-seq results from the experiments (mean ± SD, *n* = 3, One-way ANVOA). ***p < 0.001. Significantly DEGs were examined with |log2FC|>=1 & q < 0.05 was set as the threshold for significant differential expression

To investigate transcriptomic alterations associated with the DHEA-induced PCOS model, GCs from normal and DHEA-treated mice were subjected to (RNA-seq. Heatmap and volcano plot analyses demonstrated distinct transcriptomic profiles between the two groups (Fig. 3A and B). A total of 161 DEGs were identified, including 125 upregulated and 36 downregulated genes in DHEA-treated GCs compared with normals, indicating substantial transcriptomic remodeling associated with the PCOS phenotype. To verify the reliability of the RNA-seq data, nine representative DEGs were selected for qRT-PCR validation. The expression patterns of Icam1, Mro, Nr4a1, Vcam1, Comp, Car4, Irf7, Mxra8, and Cmtm1 were consistent with the RNA-seq results (Fig. 3C and D; Table 2), confirming the reliability and reproducibility of the transcriptomic analysis. These findings provided the basis for subsequent functional enrichment analyses of biological processes and signaling pathways associated with PCOS.

**Table 2:** Example of RNA-­seq results for DEGs

Gene ID	Gene name	Mean FPKM (Control)	Mean FPKM (T)	Log2FC	FDR	Regulated
ENSMUSG00000031849	Comp	2.112920251	35.19091	3.87131753726533	2.64523465659389e-20	up
ENSMUSG00000000805	Car4	1.123944953	0.069714708	-3.88392506835599	0.00225274076423468	down
ENSMUSG00000023034	Nr4a1	208.9057964	469.9333717	1.06887593198718	0.000587244841922257	up
ENSMUSG00000025498	Irf7	2.510755029	9.334837535	1.39163962435592	0.0240866531944229	up
ENSMUSG00000027962	Vcam1	1.31723558	4.259104519	1.69303674778757	0.0000945074311846414	up
ENSMUSG00000029070	Mxra8	11.27239176	33.85798353	1.41137244854084	0.0141987354148591	up
ENSMUSG00000037405	Icam1	7.984362598	17.43027	1.35331203119335	0.0141987354148591	up
ENSMUSG00000064036	Mro	13.10338587	29.63892375	1.31294504046838	0.000902004215318697	up
ENSMUSG00000110430	Cmtm1	0	0.159278	10.6373278760642	0.0084449325031215	up

### GO functional characteristics of DEGs induced by DHEA treatment

**Fig. 4:**
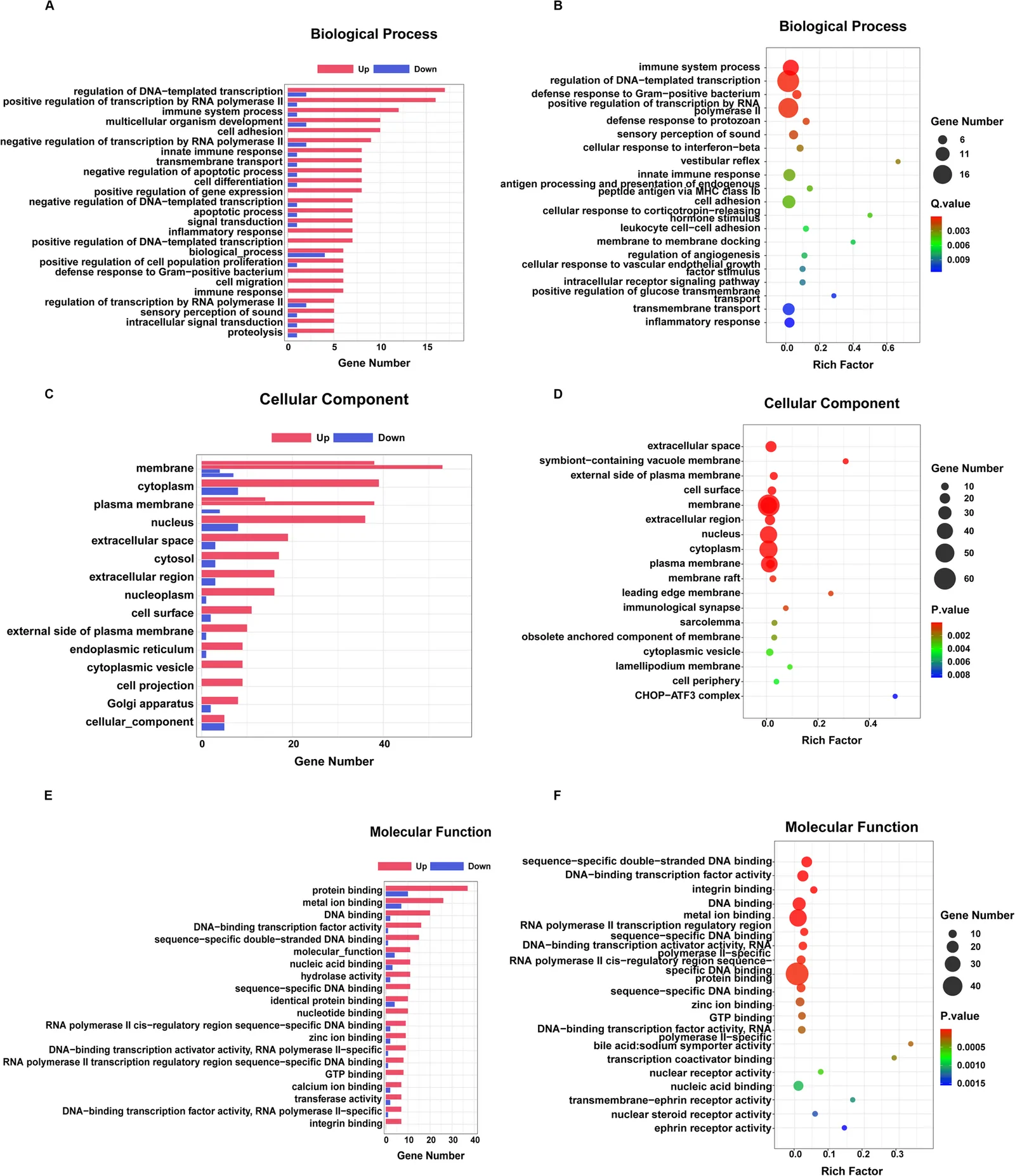
DEGs in ovary GCs were analyzed via the GO database. **A**, **B** GO enrichment analysis of DEGs in the biological process (BP) category. **C**, **D** GO enrichment analysis of DEGs in the cellular component (CC) category. **E**, **F** GO enrichment analysis of DEGs in the molecular function (MF) category

To characterize the biological functions associated with transcriptomic alterations in ovarian GCs, DEGs were subjected to GO enrichment analysis. DEGs were enriched across the biological process (BP), cellular component (CC), and molecular function (MF) categories. Among the enriched BP terms, immune system process and regulation of DNA-templated transcription were the most prominent (Fig. 4A and B). In the CC category, extracellular space and membrane-related components were significantly enriched (Fig. 4C and D), whereas protein binding, DNA binding, and nuclear steroid receptor activity were among the major enriched MF terms (Fig. 4E and F). Overall, GO analysis indicated that DHEA-induced transcriptomic alterations were primarily associated with immune-related processes, ovarian endocrine function, and steroid receptor activity, providing functional evidence for the transcriptomic changes observed in the PCOS model.

### NF-κB signaling pathway enriched in PCOS associated DEGs

**Fig. 5:**
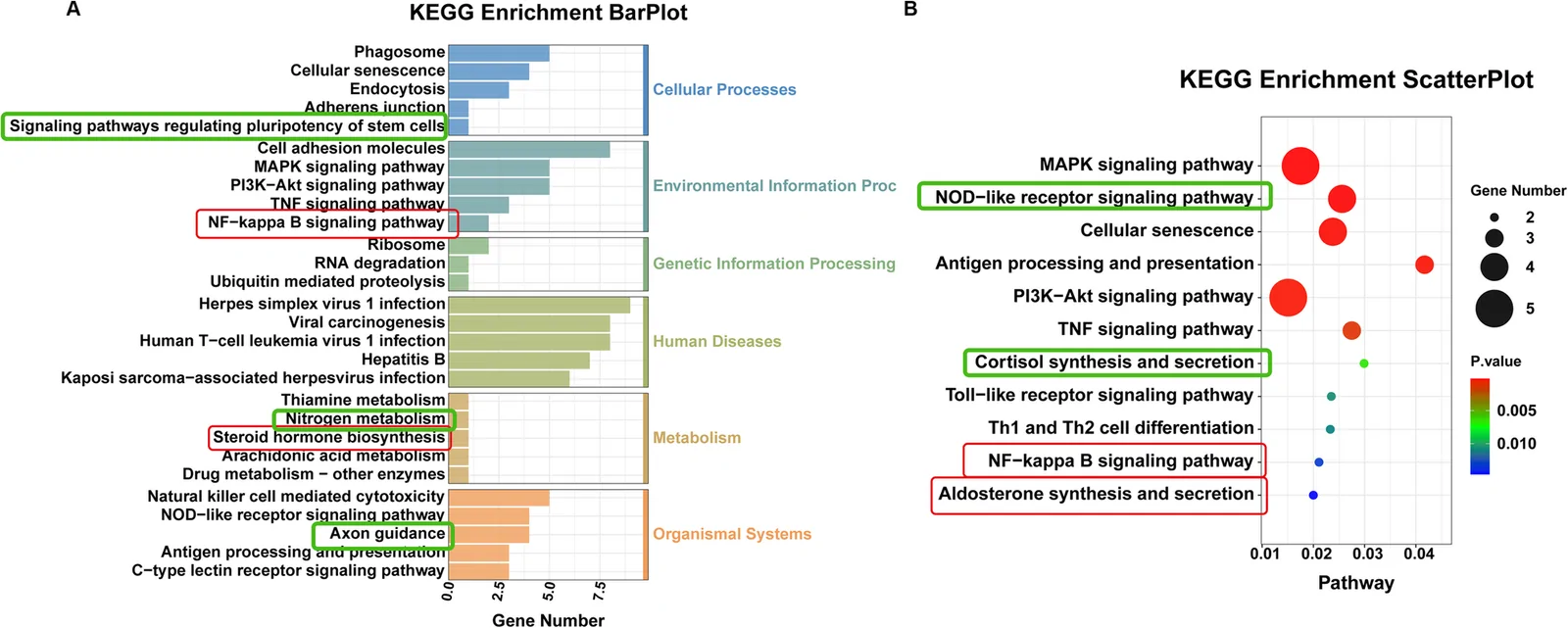
DEGs in ovary GCs analyzed via the KEGG database. **A** KEGG pathway classification and annotation of DEGs in the present study. **B** The number of DEGs in the top 13 enriched KEGG pathways

KEGG pathway enrichment analysis identified significant enrichment of pathways related to inflammation and steroid hormone metabolism in ovarian granulosa cells from DHEA-treated mice (Fig. 5A and B). The most significantly enriched pathways included the TNF signaling pathway, NF-κB signaling pathway, and steroid hormone biosynthesis pathway. Additional enriched pathways, including pathways regulating pluripotency of stem cells, nitrogen metabolism, axon guidance, NOD-like receptor signaling, and cortisol synthesis and secretion, were also identified. These results indicate that endocrine dysregulation in the DHEA-induced PCOS model was associated with broad transcriptomic alterations, particularly in inflammation- and steroidogenesis-related signaling pathways.

### Androgen supplementation regulates the expression of inflammation in the GCs in vitro

**Fig. 6:**
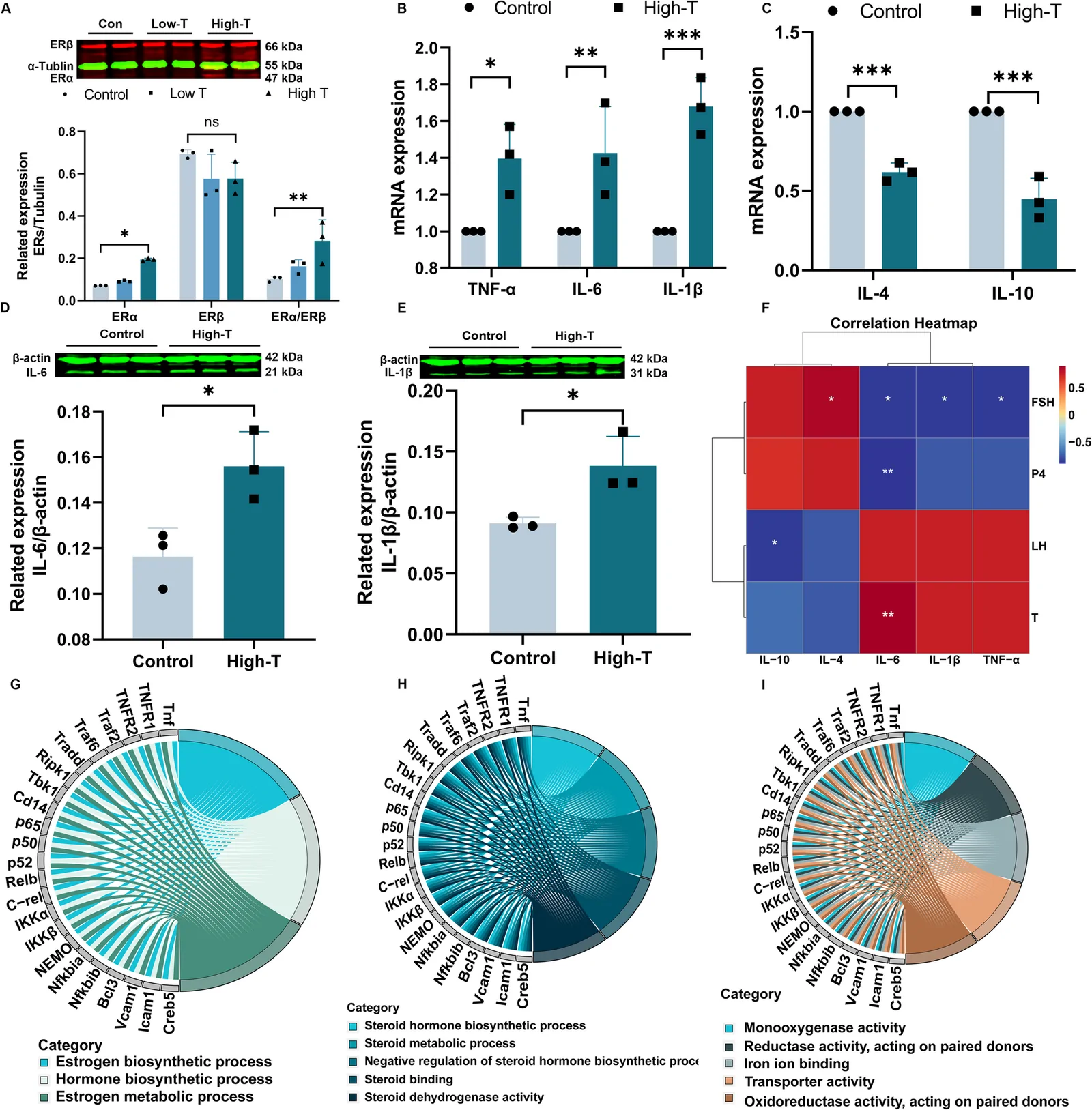
Inflammatory-related factor expression in PCOS-like GCs. **A** Changes in ERs protein expression levels in primary GCs after the addition of T (mean ± SD, *n* = 3, Two-way ANVOA). *p < 0.05, **p < 0.01. **B** The mRNA expression changes of pro‑inflammatory factors in primary GCs in response to T treatment (mean ± SD, *n* = 3, Two-way ANVOA). *p < 0.05, **p < 0.01, ***p < 0.001. **C** Changes in the mRNA expression of anti-inflammatory factors in primary GCs after the addition of T (mean ± SD, *n* = 3, Two-way ANVOA). ***p < 0.001. **D** Changes in the expression of IL-6 protein in primary GCs after the addition of T (mean ± SD, *n* = 3, T-test). *p < 0.05. **E** Changes in the expression of IL-1β protein in primary GCs after the addition of T (mean ± SD, *n* = 3, T-test). *p < 0.05. **F** Correlation heatmap between inflammatory factors and reproductive hormones. **G** Chord plot displaying selected GO terms (E2 generation) enriched of DEGs highly expressed during inflammation. **H** Chord plot displaying selected GO terms (Steroid production) enriched of DEGs highly expressed during inflammation. **I** Chord plot of selected E2 production‑associated GO terms enriched in inflammation‑driven highly expressed DEGs

To investigate the association between hyperandrogenism and inflammatory responses in ovarian GCs, primary mouse GCs were exposed to T under control (8 ng/mL) or hyperandrogenic (260 ng/mL) conditions. Compared with control cells, treatment with 260 ng/mL T significantly increased ERα expression (*P* < 0.05) and the ERα/ERβ ratio (*P* < 0.01), whereas ERβ expression remained unchanged (*P* > 0.05, Fig. 6A). No significant changes were observed in GCs treated with 8 ng/mL T. Based on the predefined experimental criteria, GCs exposed to 260 ng/mL T for 24 h were defined as PCOS-like GCs. Compared with control GCs, PCOS-like GCs exhibited significantly increased mRNA and protein expression of the pro-inflammatory cytokines IL-6 and IL-1β, together with increased tumor necrosis factor α (TNF-α) mRNA expression (Fig. 6B, D and E). In contrast, the anti-inflammatory cytokines interleukin 4 (IL-4) and interleukin 10 (IL-10) were significantly decreased (Fig. 6C). These findings indicate that hyperandrogenic conditions were associated with a shift toward a pro-inflammatory expression profile in primary GCs.

Correlation analysis further demonstrated significant associations between inflammatory factors and reproductive hormones (Fig. 6F). IL-6 showed a positive correlation with T, whereas IL-4 was positively correlated with FSH. Conversely, IL-6, IL-1β, and TNF-α were negatively correlated with FSH, while IL-10 and IL-6 were negatively correlated with LH and P4, respectively. Functional annotation of 23 inflammation-related DEGs revealed enrichment in estrogen biosynthesis, steroid hormone biosynthesis, and iron ion binding-related processes (Fig. 6G, H and I). These observations further support an association between endocrine alterations and inflammatory responses in ovarian GCs in the DHEA-induced PCOS model.

## Discussion

PCOS is multifactorial and remains incompletely understood. The most effective approach to studying these issues involves obtaining PCOS-related data from human clinical samples. However, ethical considerations, experimental costs, and subject availability have led researchers to favor the use of other mammals for constructing PCOS models [20]. The successful development of a reliable PCOS model is essential for basic biomedical research, facilitating a systematic examination of the pathological mechanisms underlying the disorder and enabling the rational optimization of clinical treatment regimens. Due to the heterogeneous clinical presentations and multifactorial pathogenesis of PCOS, accurately replicating the full spectrum of disease hallmarks in in vitro models presents a significant challenge [19]. The PCOS mouse model induced by DHEA demonstrates a degree of concordance with the clinical characteristics of PCOS, particularly regarding hormonal level changes, ovarian morphological alterations, and metabolic abnormalities [21]. In the DHEA group, the number of cystic follicles in the ovaries of mice increased, whereas the number of corpora lutea decreased. This stagnation of the sexual cycle aligns with findings from previous studies [22, 23].

Furthermore, serum levels of T and LH in the DHEA group exhibited significant increases, corroborating the results reported by Yang Liu et al. [24]. Research indicates that reduced levels of LH/FSH and P4 serve as specific markers for PCOS [25]. Our results demonstrate that the DHEA-induced mouse model employed herein exhibits clinical features consistent with those of PCOS patients, including reproductive hormone dysregulation, validating its suitability for further investigation. However, they have generally failed to exhibit the spectrum of metabolic changes observed in women with PCOS [26], probably due to the different feedback effects on the hypothalamo-pituitary axis which regulates the production of follicle stimulating hormone (FSH) and LH, 2 master regulators of ovarian function. Therefore, although the DHEA-induced model reproduces the major reproductive and endocrine features of PCOS, it does not fully recapitulate the metabolic heterogeneity observed in women with PCOS [27]. As such, they are better viewed as hypothesis-generating observations rather than definitive conclusions. Although animal models of PCOS have their limitations, use of appropriate animal models is enabling discovery, validation, and optimization of novel biomarkers and treatments for women with PCOS. Moreover, obesity—a common feature in many PCOS patients—is not consistently observed in the DHEA model unless combined with high-fat diet protocols [28, 29]. This limits the model’s ability to fully recapitulate the metabolic spectrum of human PCOS.

There are two types of estrogen receptors (ERα and ERβ) in mammalian follicles, both of which are nuclear receptors [30]. ERα and ERβ exhibit substantial disparities in both expression pattern and tissue localization in the ovary, with fundamentally divergent biological functions. ERβ is primarily localized to GCs in the normal ovary, where it plays an indispensable role in driving follicular growth and maturation [31]. This supports that ERβ is the key estrogen receptor for normal follicular development and maturation. Hence, follicular development arrest in PCOS may be related to an abnormal increase in ERα expression or the ERα/ERβ ratio. Accordingly, we compared the expression profiles of ERα and ERβ between normal mice and DHEA-induced PCOS mice. Our results revealed that both ERα expression and the ERα/ERβ ratio were markedly upregulated in the DHEA-treated group relative to control group. This finding is in line with the aforementioned reports, further validating that our established PCOS model recapitulates the molecular expression hallmarks of PCOS and laying a solid foundation for subsequent investigations. This molecular alteration is consistent with previous clinical observations in PCOS and further supports that the DHEA-induced mouse model reproduces altered estrogen signaling reported in patients. Such receptor dysregulation is closely linked to the core clinical manifestations of PCOS, including hyperandrogenism, chronic low-grade ovarian inflammation, follicular arrest and ovulation disorders. However, the present study does not establish that altered ERα signaling directly mediates inflammatory responses.

RNA-seq has been widely and successfully applied in the field of pathological mechanism research, serving as an effective tool for identifying DEGs and regulatory networks under specific conditions [32]. For instance, numerous independent studies have performed RNA‑seq on oocytes and cumulus cells obtained from PCOS patients and matched fertile healthy controls [33]. To date, multiple transcriptomic analyses have been performed to explore molecular alterations in PCOS ovarian tissues, GCs and hyperandrogenic cell models. Consistent with these published studies, our RNA-seq results also identified a large set of DEGs enriched in classic inflammatory signaling (NF-κB, TNF), steroid hormone metabolism and follicle development pathways. While these findings confirm previously reported inflammatory and steroidogenesis-related pathways, our study further integrates GC transcriptomic alterations with endocrine changes and inflammatory responses in the same experimental model. The dysregulation of inflammatory mediators and hormone-related genes observed in our dataset further validates the universal molecular characteristics of hyperandrogenism-induced ovarian dysfunction across different models. Collectively, the overlapping findings demonstrate the reliability and reproducibility of our transcriptomic data. Beyond the well-known pathways, our GO and KEGG enrichment analyses uncovered several previously underreported signaling cascades, pathways regulating pluripotency of stem cells, nitrogen metabolism, axon guidance, the NOD-like receptor pathway, and cortisol synthesis and secretion, that are rarely highlighted in existing PCOS transcriptomic studies. Our work therefore not only confirms existing molecular signatures, but also provides additional candidate genes for future mechanistic studies.

By aligning RNA-seq data to a well-annotated genome, we precisely quantified differential gene expression in PCOS mice, and revealed candidate genes, such as Comp, Car4, Irf7, Mxra8, and Cmtm1 [34, 35]. These genes have been rarely investigated in PCOS. Functionally, they participate in complement response, interferon signaling, steroid metabolism, cell adhesion and chemokine regulation, respectively. These candidate genes may contribute to the transcriptomic alterations observed in PCOS and warrant further functional investigation. Recent in silico transcriptomic studies using weighted gene co-expression network analysis have similarly identified dysregulated long non-coding RNAs and gene modules as potential PCOS-associated markers [36]. Only transcriptional expression of key DEGs was validated by qRT-PCR, while their specific biological functions were not experimentally characterized. The exact mechanisms by which Comp, Car4, Irf7, Mxra8 and Cmtm1 participate in PCOS-related phenotypes remain to be elucidated. In addition, the conclusions are drawn from an in vitro cell model, and further in vivo and clinical sample validation is necessary. We will perform functional investigations of these candidate genes in our follow-up work.

Mounting clinical data confirm that circulating concentrations of inflammatory biomarkers and the expression of their cognate genes are significantly elevated in patients diagnosed with PCOS. Notably, circulating levels of CRP, IL‑18, TNF‑α, and IL‑6 are significantly increased in patients with PCOS compared with healthy controls matched for age and body mass index (BMI) [37–39]. In a clinical investigation, Deligeoroglou et al. documented markedly increased concentrations of IL‑18, MCP‑1, and MIP‑1α with PCOS relative to healthy control subjects [40–44]. Recent evidence from peripheral blood mononuclear cells has further suggested that systemic immune dysregulation and persistent inflammatory biomarker expression accompany PCOS [45]. However, most studies on PCOS-related inflammation have focused on serum or endometrial samples rather than the ovary and follicles, with limited research investigating the expression of inflammatory cytokines and inflammasomes in PCOS patient-derived GCS. Therefore, the present study analyzed inflammatory factor levels in T-treated GCs. Prior to this study, we treated mouse ovarian GCs with 260 ng/mL T, which was validated as a PCOS-like model due to upregulated ERα expression and an increased ERα/ERβ ratio, which is consistent with the molecular markers of in vivo PCOS models. Our results show that TNF, IL-6, and IL-1β levels are increased in PCOS-like GCs, suggesting that hyperandrogenic conditions are associated with enhanced inflammatory responses in ovarian GCs. Notably, TNF, IL-6, and IL-1β are essential regulators of ovulatory processes; dysregulation of these inflammatory mediators contributes to impaired ovulation, ranging from ovulatory dysfunction to overt anovulation [46]. The inflammatory profile observed in T-treated GCs was consistent with that reported in PCOS animal models and clinical studies. Since this concentration [47] did not cause cytotoxicity, the inflammation is unlikely due to non-specific pharmacological stress. The inflammatory signature is consistent with PCOS-related ovarian inflammation reported in clinical samples and animal models, supporting its relevance to PCOS-associated ovarian inflammatory alterations. In future studies, we will construct cell models with gene knockdown or overexpression to clarify the roles of these genes in regulating ovarian inflammation and follicle function. In addition, we will verify our findings using PCOS animal models and clinical samples.

Although inflammation and reproductive hormone dysregulation are two core features of PCOS, their interplay remains incompletely elucidated [48]. Mounting evidence suggests that supraphysiological androgen levels are associated with chronic low-grade inflammation in patients with PCOS [48–50]. Our results further demonstrate an association between reproductive hormone dysregulation and inflammatory alterations in ovarian GCs. Conversely, other studies have proposed that persistent inflammation is the primary driver of androgen overproduction and underpins ovarian dysfunction in PCOS patients [51]. The relationship between endocrine and immune abnormalities in PCOS is likely influenced by additional genetic and environmental factors. Recent meta-analyses have suggested that vitamin D receptor gene polymorphisms may be associated with susceptibility to PCOS and could potentially influence endocrine and inflammatory phenotypes [52]. These findings indicate that the hormone–inflammation association observed in our study may form part of a broader network involving genetic susceptibility, immune regulation, and ovarian steroid metabolism. Nevertheless, VDR signaling was not examined in the present experiments, and its relevance to the identified GC transcriptomic changes remains to be established.

This study has several limitations. First, the RNA-seq findings were validated by qRT-PCR but require independent validation in additional animal cohorts and clinical samples. Second, the relatively small sample size may limit statistical power. Third, although the DHEA-induced mouse model reproduces major reproductive features of PCOS, it cannot fully reflect the metabolic heterogeneity of human PCOS, and species-specific differences should be considered when interpreting the findings. Finally, the present study identifies associations between endocrine dysregulation and inflammatory responses rather than direct causal relationships. Future mechanistic studies are needed to determine the biological significance of these associations.

## Conclusions

In conclusion, this study established and validated a DHEA-induced mouse PCOS model and an in vitro PCOS-like granulosa cell (GC) model to investigate GC dysfunction under hyperandrogenic conditions. Integrated transcriptomic and experimental analyses revealed that ovarian GCs from PCOS mice exhibited extensive gene expression remodeling, with enrichment of inflammation- and steroid hormone biosynthesis-related pathways. Furthermore, T-treated primary GCs displayed a pro-inflammatory expression profile, and correlation analysis indicated an association between reproductive hormone dysregulation and inflammatory alterations in ovarian GCs. These findings provide new insights into the relationship between endocrine abnormalities, GC transcriptomic changes, and inflammation in PCOS. The established PCOS-like GC model and identified candidate pathways may serve as valuable resources for future mechanistic studies. Further investigations using functional approaches and human clinical samples are required to determine the causal relationships underlying these associations and to evaluate their translational relevance.

## Supplementary Information

Supplementary Material 1.

## Data Availability

The datasets generated and/or analysed during the current study are available in the GEO repository, GSE315837.

## Abbreviations

PCOS: Polycystic ovary syndrome
GCs: Granulosa cells
DHEA: Dehydroepiandrosterone
ERs: Estrogen receptors
ELISA: Enzyme-linked immunosorbent assay
T: Testosterone
LH: luteinizing hormone
FSH: Follicle-stimulating hormone
RNA-seq: RNA sequencing
DEGs: Differentially expressed genes
GO: Gene Ontology
KEGG: Kyoto Encyclopedia of Genes and Genomes
Icam1: Intercellular adhesion molecule 1
Mro: Maestro
Nr4a1: Nuclear receptor subfamily 4 group A member 1
Vcam1: Vascular cell adhesion molecule 1
Comp: Cartilage oligomeric matrix protein
Car4: Marbonic anhydrase 4
Irf7: Interferon regulatory factor 7
Mxra8: Matrix remodeling associated 8
Cmtm1: KLF like marvel transmembrane domain containing 1
IL-6: Inflammatory markers interleukin 6
IL-1β: Interleukin 1β
TNF-α: Tumor necrosis factorα
IL-4: Interleukin 4
IL-10: Interleukin 10
