# Supplementary material for: Association of ovarian inflammation and hormonal dysregulation: a candidate mechanism underlying polycystic ovary syndrome
Source: J Ovarian Res. 2026 Aug 4;19:329. doi: 10.1186/s13048-026-02218-3 (PMC13721681; doi:10.1186/s13048-026-02218-3)
Supplement: Supplementary file 1 [file 13048_2026_2218_MOESM1_ESM.pdf]

This document includes all original, unprocessed images corresponding to the cropped blots/gels presented in the main manuscript (e.g., Western blots for Estrogen receptor α, Estrogen receptor β, IL-6, IL-1β, and loading controls such as α-Tubulin and β-actin; SDS-PAGE gels for protein separation verification). No post-acquisition processing (e.g., brightness modification, or pixel manipulation) has been performed on these images, except for the addition of sample labels and molecular weight markers (as required for clarity).

We apologize for the incomplete regarding the partial marker display and would like to clarify that this was a deliberate and scientifically justified choice to optimize data presentation, without compromising the accuracy or integrity of the results. Below is a detailed explanation and supplementary measures to address this concern:

1.Rationale for Partial Marker Display

The target proteins in our study (e.g., estrogen receptor α: ~47 kDa, and estrogen receptor β: ~66 kDa, IL-6: ~21 kDa, IL-1β: ~31 kDa, α-Tubulin: ~55 kDa, β-actin: ~42 kDa) have well-characterized molecular weights, as reported in numerous peer-reviewed studies[1-3] and confirmed by the antibody datasheets and Uniprot.

To enhance the visibility of the target bands and avoid unnecessary visual clutter, we only displayed the relevant MW marker bands corresponding to the target protein range (e.g., 45–70 kDa for estrogen receptor α, estrogen receptor β and α-Tubulin, 15–55 kDa for IL-6, IL-1β, and β-actin) in the gel. The excluded marker bands (e.g., <15 kDa or >70 kDa) were irrelevant to the target proteins and would not aid in band identification.

2. Verification of Band Accuracy

The identity of each target band was confirmed through multiple complementary methods, ensuring no misinterpretation:

(1) We are using [PM2510, SMOBIO] ExcelBand™ Enhanced 3-color Regular Range Protein Marker (9-180 kDa) in Figure S1A-S1D, [26616, ThermoFisher] PageRuler™ pre-stained protein molecular weight standard (10 to 180 kDa) in Figure S1E, the size of the bands can be clearly identified based on the color of the gel and can be cut accordingly.

(2) Loading control reference: α-Tubulin (MW ~55 kDa) and β-actin(MW ~42 kDa) was used as a loading control, and its consistent migration across all lanes (aligned with the 55 kDa and 42 kDa marker band) confirmed the reliability of the electrophoresis and transfer process.

We confirm that the partial marker display does not affect the validity or interpretation of the Western blot results. The multi-faceted validation ensure full transparency and data reliability.

Figure S1A

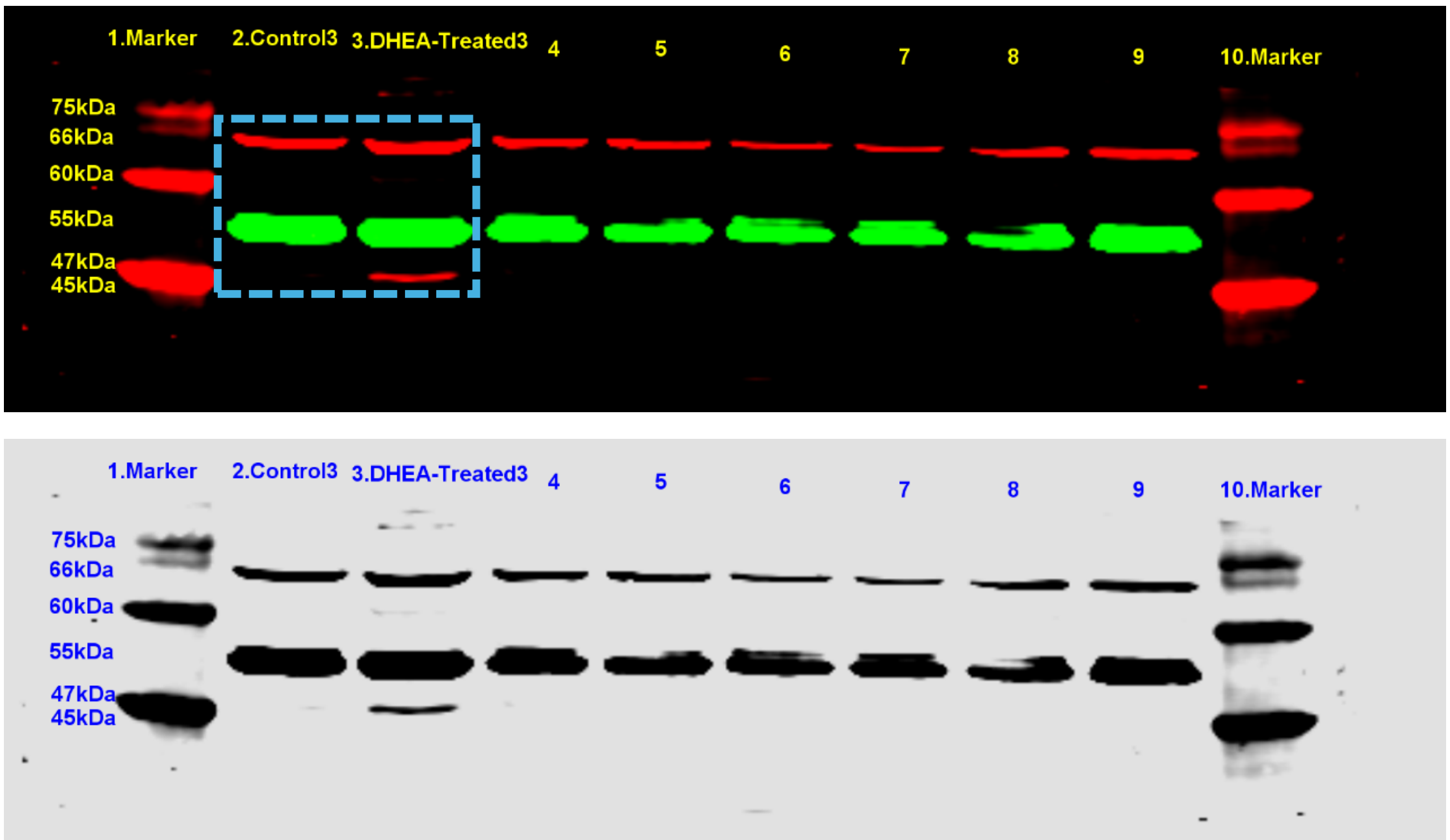

Figure S1A Western blot analysis of estrogen receptor α and estrogen receptor β in ovarian granulosa cells of mice with PCOS induced by DHEA.

4-9: Sample Control, DHEA treated(duplicate, poor result).

Experimental conditions:

Sample source: PCOS mice induced by DHEA ovarian granulosa cell;

Treatment concentrations: DHEA group animals received daily injections of DHEA (6 mg/100 g body weight, 0.1 mL). An equal volume of absolute ethanol and sesame oil mixture to the control group;

Electrophoresis parameters: 12% SDS-PAGE gel (separating gel) and 4% stacking gel; constant voltage (80 V for stacking gel, 120 V for separating gel) for 90 min; wet transfer (200 mA, 90 min) to PVDF membrane (0.45 μm, Millipore);

Antibody information: Primary antibodies—rabbit anti-ERβ antibody (Thermo Fisher Scientific, PA1-311, 1:1000 dilution), rabbit anti-ERα antibody (Thermo Fisher Scientific, PA5-16440, 1:1000 dilution), mouse anti-α-tubulin antibody (Sigma, T5168, 1:10,000 dilution); secondary antibodies—specifically donkey anti-rabbit (LI-COR Biosciences, 926-32213, 1:10,000 dilution) and donkey anti-mouse (LI-COR Biosciences, 926-32212, 1:10,000 dilution); incubation conditions: Primary antibodies at 4°C overnight, secondary antibodies at room temperature for 1 h.

Note: This image corresponds to the cropped blot in Figure 2F of the main manuscript. The target bands (**estrogen receptor α: ~47 kDa, and estrogen receptor β: ~66 kDa, α-Tubulin: ~55 kDa**) are indicated by red arrows. The exact position of the cropped region used in Figure 2F of the main manuscript is highlighted with a dashed box.

The **α-Tubulin** expression across all lanes to confirm equal protein loading.

Figure S1B

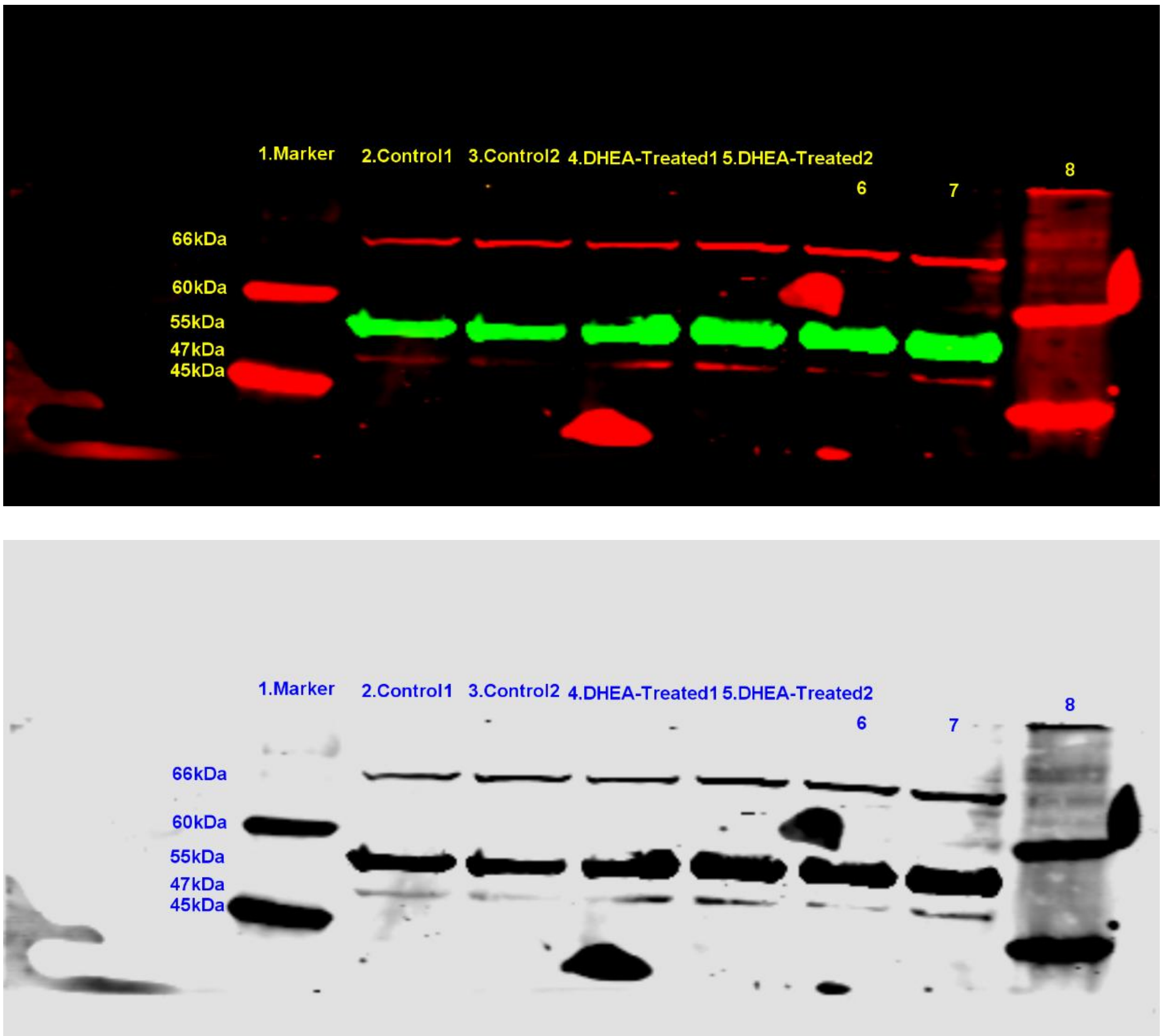

Figure S1B Western blot analysis of estrogen receptor α and estrogen receptor β in ovarian granulosa cells of mice with PCOS induced by DHEA.

Figure S1B and Figure S1A together constitute three biological replicates, with the experimental conditions being the same.

6-7: Unrelated sample.

8: Marker

Figure S1C

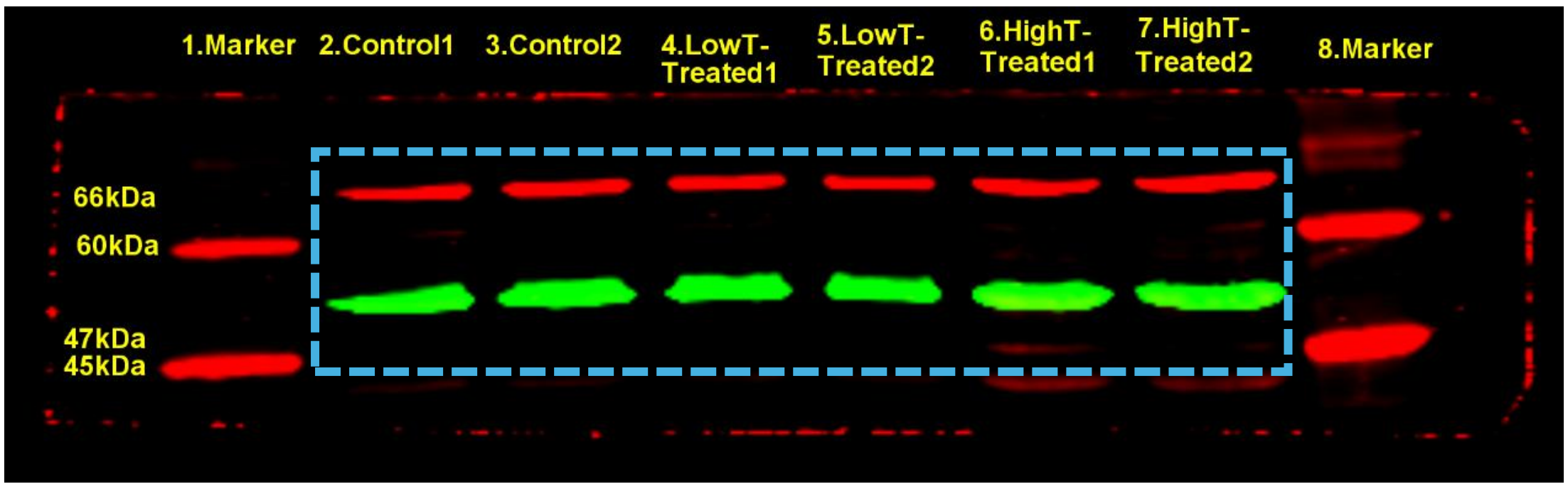

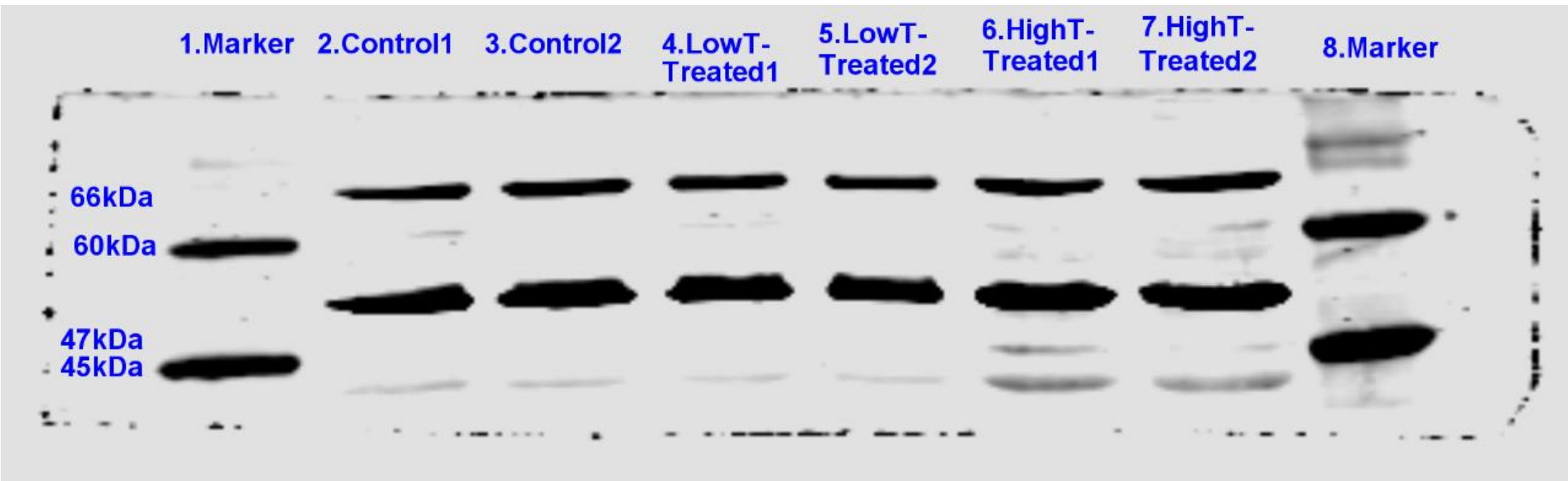

Figure S1C Western blot analysis of estrogen receptor α and estrogen receptor β in primary granulosa cells of mice were cultured and T was added.

Experimental conditions:

Sample source: mouse ovarian primary granulosa cells treated with T;

Treatment concentrations: After 24 h of in vitro culture, normal F1 GCs were treated with T (Sigma, St. Louis, MO, America) and harvested 24 h thereafter. Based on our laboratory's previous work, two T concentrations (8 ng/mL [low T] and 260 ng/mL [high T]) were used in the experiment;

Electrophoresis parameters: 12% SDS-PAGE gel (separating gel) and 4% stacking gel; constant voltage (80 V for stacking gel, 120 V for separating gel) for 90 min; wet transfer (200 mA, 90 min) to PVDF membrane (0.45 μm, Millipore);

Antibody information: Primary antibodies—rabbit anti-ERβ antibody (Thermo Fisher Scientific, PA1-311, 1:1000 dilution), rabbit anti-ERα antibody (Thermo Fisher Scientific, PA5-16440, 1:1000 dilution), mouse anti-α-tubulin antibody (Sigma, T5168, 1:10,000 dilution); secondary antibodies—specifically donkey anti-rabbit (LI-COR Biosciences, 926-32213, 1:10,000 dilution) and donkey anti-mouse (LI-COR Biosciences, 926-32212, 1:10,000 dilution); incubation conditions: Primary antibodies at 4°C overnight, secondary antibodies at room temperature for 1 h.

Note: This image corresponds to the cropped blot in Figure 6A of the main manuscript. The target bands (**estrogen receptor α: ~47 kDa, and estrogen receptor β: ~66 kDa, α-Tubulin: ~55 kDa**) are indicated by red arrows. The exact position of the cropped region used in Figure 6A of the main manuscript is highlighted with a dashed box.

The **α-Tubulin** expression across all lanes to confirm equal protein loading.

Figure S1D

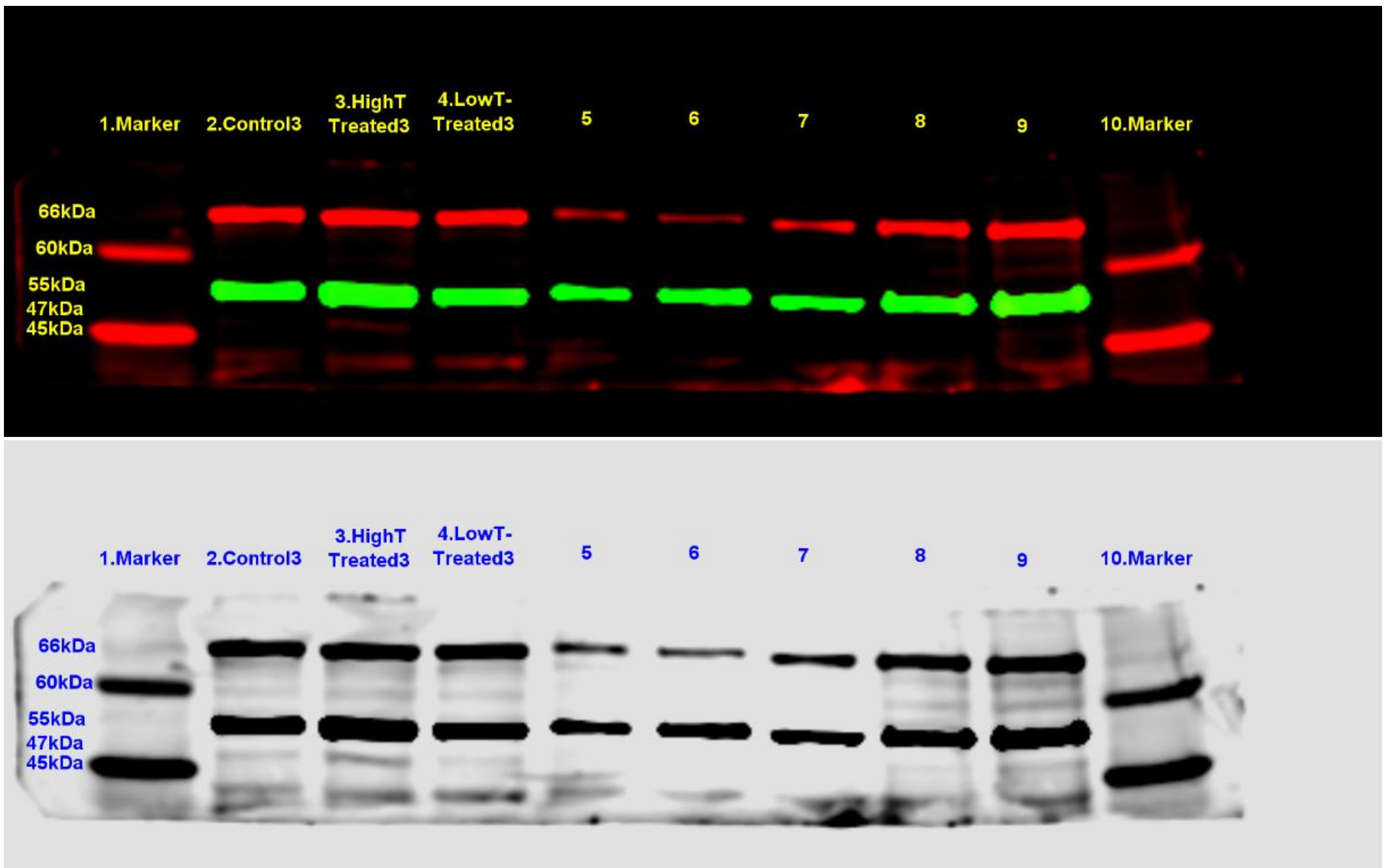

Figure S1D Western blot analysis of estrogen receptor α and estrogen receptor β in primary granulosa cells of mice were cultured and T was added.

5 and 9: Unrelated sample.

6-8: Sample Control, HighT Treated, LowT Treated(duplicate, poor result).

Figure S1C and Figure S1D together constitute three biological replicates, with the experimental conditions being the same.

Figure S1E

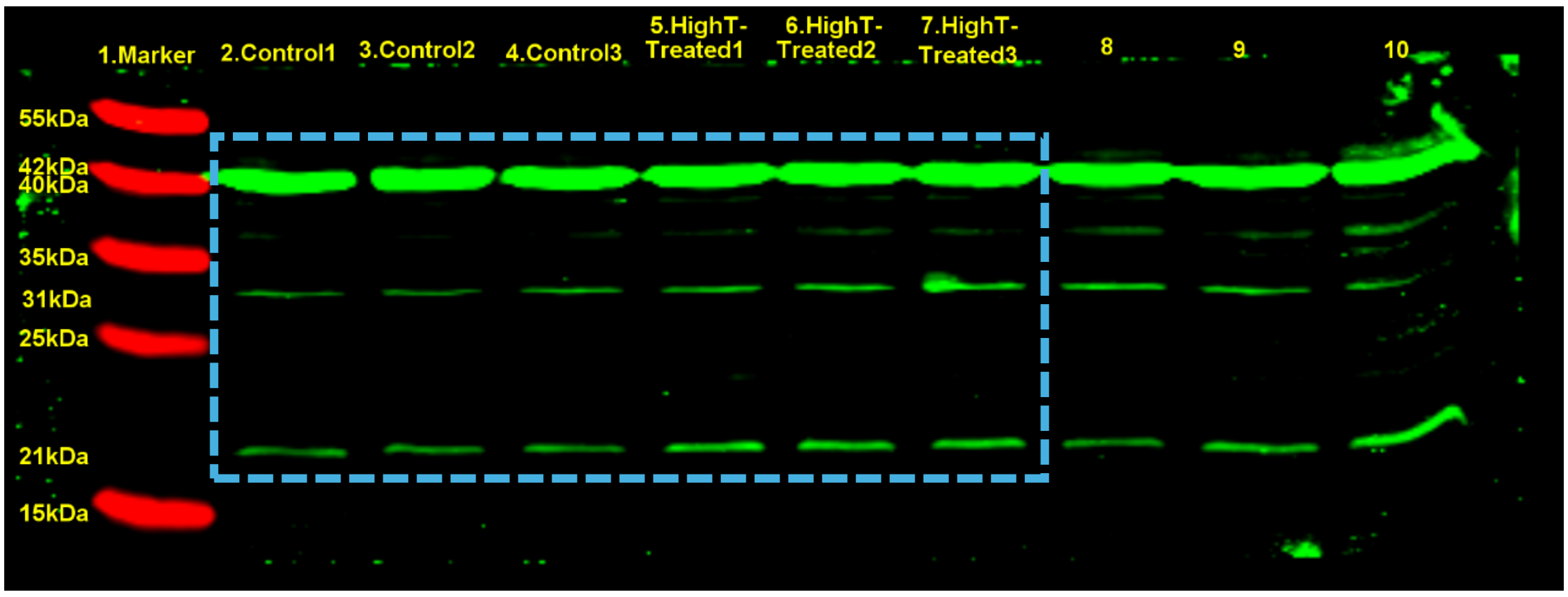

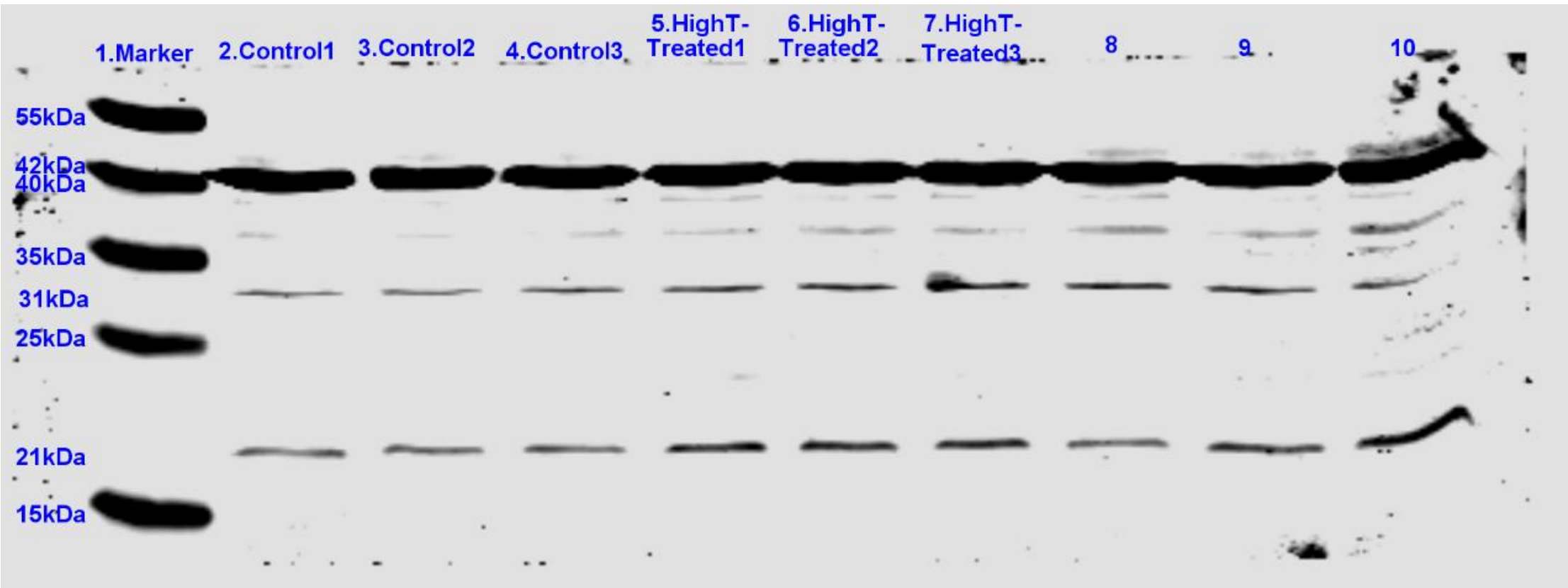

Figure S1E Western blot analysis of IL-6 and IL-1β in primary granulosa cells of mice were cultured and T was added.

8-10: Unrelated sample.

Experimental conditions:

Sample source: mouse ovarian primary granulosa cells treated with T;

Treatment concentrations: After 24 h of in vitro culture, normal F1 GCs were treated with T (Sigma, St. Louis, MO, America) and harvested 24 h thereafter. Based on our laboratory's previous work, 260 ng/mL [high T]) were used in the experiment;

Electrophoresis parameters: 12% SDS-PAGE gel (separating gel) and 4% stacking gel; constant voltage (80 V for stacking gel, 120 V for separating gel) for 90 min; wet transfer (200 mA, 90 min) to PVDF membrane (0.45 μm, Millipore);

Antibody information: Primary antibodies—mouse anti-IL-1β (Huaan, HA601036, 1:1000), and mouse anti-IL-6 (Huaan, EM1701-45, 1:500), mouse anti-β-actin antibody (Sigma, A5316, 1:10,000); secondary antibodies—specifically donkey anti-mouse (LI-COR Biosciences, 926-32212, 1:10,000 dilution); incubation conditions: Primary antibodies at 4°C overnight, secondary antibodies at room temperature for 1 h.

Note: This image corresponds to the cropped blot in Figure 6D-6E of the main manuscript. The target bands (**IL-6: ~21 kDa, and IL-1β: ~31 kDa, β-actin: ~42 kDa**) are indicated by red arrows. The exact position of the cropped region used in Figure 6D-6E of the main manuscript is highlighted with a dashed box.

The **β-actin** expression across all lanes to confirm equal protein loading.

[1] XU X L, HUANG Z Y, YU K, et al. Estrogen Biosynthesis and Signal Transduction in Ovarian Disease [J]. Front Endocrinol (Lausanne), 2022, 13(827032.

[2] HEWITT S C, KORACH K S. Estrogen Receptors: New Directions in the New Millennium [J]. Endocr Rev, 2018, 39(5): 664-75.

[3] MAC GIOLLABHUI N, NG T H, ELLMAN L M, et al. The longitudinal associations of inflammatory biomarkers and depression revisited: systematic review, meta-analysis, and meta-regression [J]. Mol Psychiatry, 2021, 26(7): 3302-14.
